# Deep Spatial-Temporal Joint Feature Representation for Video Object Detection

**DOI:** 10.3390/s18030774

**Published:** 2018-03-04

**Authors:** Baojun Zhao, Boya Zhao, Linbo Tang, Yuqi Han, Wenzheng Wang

**Affiliations:** 1 School of Information and Electronics, Beijing Institute of Technology, Beijing 100081, China; zbj@bit.edu.cn (B.Z.); zhaoboya@bit.edu.cn (B.Z.); yuqi_han@bit.edu.cn (Y.H.); wwz@bit.edu.cn (W.W.); 2 Beijing Key Laboratory of Embedded Real-time Information Processing Technology, Beijing Institute of Technology, Beijing 100081, China

**Keywords:** deep neural network, video object detection, temporal information, Siamese network, multiscale feature representation

## Abstract

With the development of deep neural networks, many object detection frameworks have shown great success in the fields of smart surveillance, self-driving cars, and facial recognition. However, the data sources are usually videos, and the object detection frameworks are mostly established on still images and only use the spatial information, which means that the feature consistency cannot be ensured because the training procedure loses temporal information. To address these problems, we propose a single, fully-convolutional neural network-based object detection framework that involves temporal information by using Siamese networks. In the training procedure, first, the prediction network combines the multiscale feature map to handle objects of various sizes. Second, we introduce a correlation loss by using the Siamese network, which provides neighboring frame features. This correlation loss represents object co-occurrences across time to aid the consistent feature generation. Since the correlation loss should use the information of the track ID and detection label, our video object detection network has been evaluated on the large-scale ImageNet VID dataset where it achieves a 69.5% mean average precision (mAP).

## 1. Introduction

Object detection in images has received much attention in recent years with tremendous progress mostly due to the emergence of deep neural networks, especially deep convolutional neural networks [1,2,3,4], and their region-based descendants [5,6,7,8,9,10,11]. These methods achieve excellent results on still image datasets, such as Pattern Analysis, Statistical Modelling and Computational Learning Visual Object Classes (PASCAL VOC) and Microsoft Common Object in Context (COCO).

With this success, computer vision tasks have been extended from the still image domain to the video domain because, in reality, the data sources of practical applications, such as smart surveillance, self-driving, and face recognition, are mostly videos. Thus, the additional challenges are [12]: (1) motion blur: due to rapid camera or object movement; (2) quality: due to the quality of internet video clips being lower than that of still images, even if the resolutions are the same; (3) partial occlusion: due to the position change in the camera or object; and (4) pose: due to unconventional object-to-camera poses that are frequently shown in video clips. To overcome this gap, most video object detection methods [13,14,15,16] use exhaustive post-processing in addition to still image detectors. For example, T-CNN [13] uses the two-stage Faster RCNN [8] detection framework for individual video frames. Then, context suppression and tracking are applied for the detection results. Since they do not actually involve temporal information, those video object detection methods do not have favourable results on video sources.

In this paper, to introduce time-series information to the network, we propose a novel training framework based on the Siamese network [17,18], which is fully-convolutional with respect to adjacent frames. By introducing the Siamese network, we can effectively maintain feature consistency by computing the feature similarity across adjacent frames, which maintains feature consistency in the detection process. In the training stream, the input is a pair of neighboring frames. Then, a backbone network and a set of fully-convolutional layers are cascaded to generate the multiscale feature representation for detection. In this way, we can handle multiscale objects. Afterwards, we design a novel loss function based on the Siamese network to maintain the feature consistency. Correlation loss is a constraint between neighboring frames that is calculated by computing the similarity of the object features with the same label and track ID. In the detection stream, by sharing with the multiscale feature representation, the detection results are optimized by Soft-NMS [19].

Our network is evaluated on the larger-scale ImageNet VID validation dataset. Our proposed network has a 1.6% improvement compared to the baseline Single Shot Multibox Detector. Moreover, for demonstrate the effectiveness of the proposed framework, we also conduct an experiment on the YouTube Object dataset and the proposed framework has a 4.4% bonus compared to the baseline.

## 2. Related Works

In this section, we review still image object detection, video object detection, video action detection, and 3D shape classification.

### 2.1. Still Image Object Detection

There are two classes of detection methods. One method is based on region proposal classification, and the other method is based on sliding windows. Before the larger improvement of computational sources, the best of these two approaches are the Deformable Part model (DPM) [20] and Selective Search (SS) [21]. After the dramatic improvement that was shown by R-CNNs [8], region proposal object detection methods became prevalent. For the one-stage detection framework based on sliding windows, the You Look Only Once (YOLO) [22] and Single Shot Multibox Detector (SSD) [10] became renowned. These two detection methods have comparable results. Moreover, there are added benefits to using the one-stage architecture. The processing speed of the one-stage method is faster than the region proposal method. For example, SSD300 obtained a speed of 46 fps, whereas the Faster RCNN [6] was 7 fps.

### 2.2. Video Object Detection

Recently, ImageNet [23] introduced a new solution for object detection from video clips, which is the well-known ImageNet VID. This competition brings the object detection task into the video domain. In this competition, nearly all the detection methods that involve time-series information have been post-processed after the detection on individual frames. The T-CNN [13] incorporates optical flow information to fix the neighboring frame results. The MCMOT [16] approaches post-processing as a multi-object tracking problem by applying a series of man-made rules (e.g., the threshold of confidence and changing point detection). The Seq-NMS [14] regards post-processing as a confidence re-scoring problem. The boxes of the video sequence are re-scored to the average confidence. Unfortunately, these methods incorporate temporal information by post-processing and the detection in a video is a multi-stage pipeline. The temporal information is not truly involved in the algorithm.

### 2.3. Video Action Detection

Different from video object detection, video action detection is to detect every occurrence of a given action within a long video, and to localize each detection both in space and time. How to use the temporal information effectively is the common problem in video object detection and video action detection. Finding Action Tubes [24] extracts frame-level action proposals using selective search and link them using Viterbi algorithm. Multi-region RCNNs [25] applies two stream R-CNNs for action detection, where a spatial Region Proposal Network (RPN) and a motion RPN are used to generate frame-level action proposals. Tube CNN [26] proposes an end-to-end Tube Convolutional Neural Network for action detection, which exploits a 3D convolutional network to extract spatial-temporal features.

### 2.4. 3D Shape Classification

3D data has three-dimensional information, which consists of 2D information and depth information. A video clip is similar to 3D data, which also has three-dimensional data (2D information and time-series information). The processing of 3D data is mainly focused on shape classification. The common problem of video object detection and 3D shape classification is how to use the third-dimensional information (depth or time-series information). LWU [27] leverages stochastic gradient Markov Chain Monte Carlo (SG-MCMC) and Bayesian interpretations to learn the weight uncertainty and improve the robustness in deep neural networks. GM3D [28] extracts geodesic moments as shape features and uses a two-layer stacked sparse auto encoder to digest these features to predict the shape category.

## 3. Method

**Figure 1:**
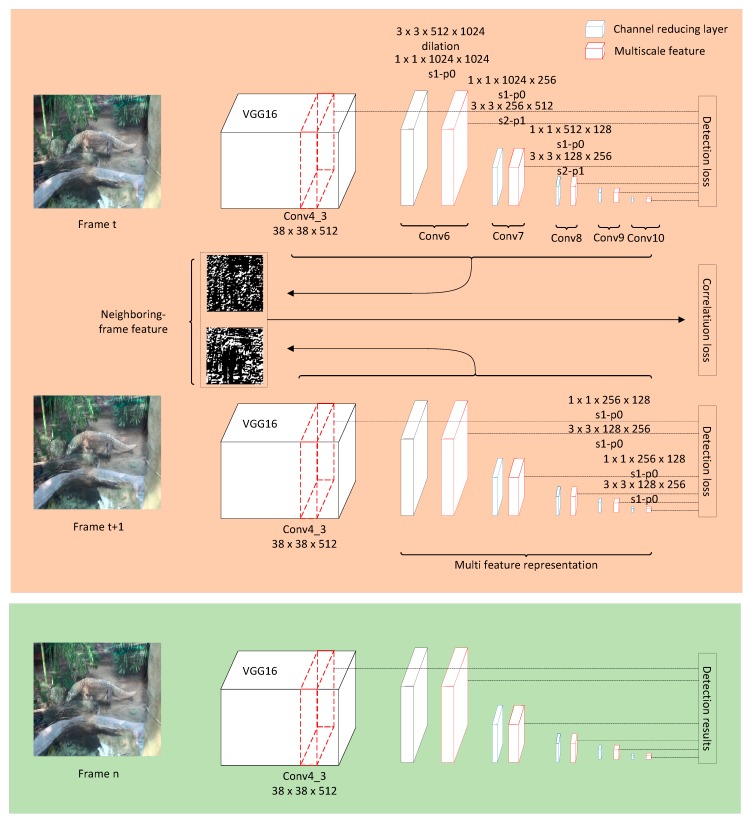
The architecture of the proposed method. The orange part is the training procedure by neighboring frames. The green part is the testing procedure of frame *n*.

Figure 1 illustrates the entire pipeline of the proposed method. The pipeline is introduced in two parts: the training stream and the testing stream. First, for the training procedure to have a better training starting point, the backbone of the proposed fully-convolutional neural networks is the VGG-16, which is pre-trained by using the ImageNet CLS-LOC dataset. Second, by feeding forward a set of fully-convolutional layers cascaded after the backbone network, the multiscale feature representation is generated to handle multiscale objects. Third, a set of different anchor shapes are generated on the multiscale feature maps to adapt objects with different scales’ aspect ratios. Fourth, considering the predictions for each anchor and the intersection of union (IOU) of the anchor and ground truth, the detection loss is formed. Finally, the above procedures are replicated twice to establish a Siamese network. By measuring the similarity with the neighboring frame feature, the correlation loss, which consists of the center-value loss and the anchor coordinate loss, is computed. For the testing procedure, after sharing the backbone multiscale feature representation, anchor generation, and prediction, the testing results are computed after the Soft-NMS optimization.

### 3.1. Network Architecture

The following section details the proposed network, which contains the backbone network, multiscale feature representation, anchor generation, anchor prediction, and training sample selection. The network architecture explains the data flow in the feed-forward procedure.

#### 3.1.1. Backbone Network

**Figure 2:**
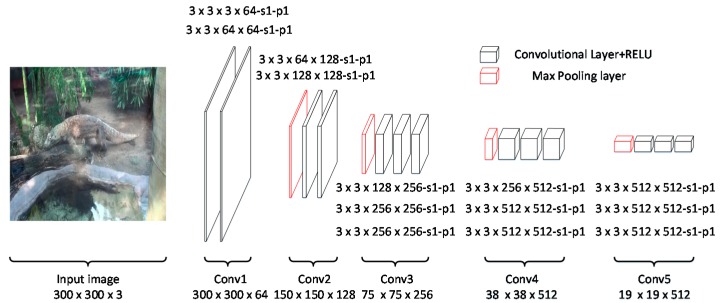
The architecture of VGG16 that showing the convolutional and pooling layers. Along with the feed-forward procedure, the size of the feature map is decreased. *s* and *p* refer to stride and padding size, respectively.

In this paper, the backbone network is the VGG-16 [3], which is pre-trained on the ImageNet CLS-LOC dataset [23]. As shown in Figure 2, the original VGG-16 is a deep CNN that includes 13 convolutional layers and three fully-connected layers. The convolutional layers generate deep features, which are then fed into the fully-connected layers. Similar to SSD, we remove the fully-connected layers and only use the convolutional layers to generate the feature maps.

The followings detail the entire backbone network:

(1) Input: images with RGB channels.
(2) Convolutional layers: The convolutional layers mainly contain five groups—conv1, conv2, conv3, conv4, and conv5. Conv1 includes two convolutional layers with 64 3×3 kernels. Conv2 includes two convolutional layers with 3×3 kernels. Similar to conv1 and conv2, conv3, conv4, and conv5 include three convolutional layers with 256, 512, and 512 3×3 kernels, respectively.
(3) The activation function is rectified linear units [29], and the kernel size of the max pooling layer is 2×2.

#### 3.1.2. Multiscale Feature Representation

After the backbone network, the multiscale feature representation network is cascaded, which is generated by feed-forward convolutional networks [4,30]. According to SSD [10], conv4_3 has a different feature scale compared to the other layers in the backbone network. Therefore, the multiscale feature representation is started from conv4_3 after applying a L2 normalization to scale the feature norm at each location in the feature map to 20 and to learn the scale during back propagation.

Considering that the size of the input image is initialized to 300×300, conv4_3 has a 38×38 feature map size, and conv4_3 is the first feature scale map. The multiscale feature representation is generated after conv4_3 by applying feed-forward convolution.

Traditionally, the low-resolution feature map can be generated from the high-resolution feature map by a certain convolutional layer, but the computational costs are high. To reduce the computational costs, each scale block contains 2 convolutional layers. The first convolutional kernel size is 1×1×M×N. Here, *M* is the upper layer channel number, and *N* is the current channel number. By using a 1×1×M×N kernel, the middle feature map channel is decreased from *M* to *N*. The second convolution kernel size is 3×3×N×K, where *K* is the current scale-feature channel number.

Table 1 shows the details of the multiscale feature representation. There are six scale feature maps with feature map sizes of 38×38, 19×19, 10×10, 5×5, 3×3, and 1×1, where 38×38 is the conv4_3 in VGG16. In the options list, *s* is the convolutional kernel stride, *p* is the padding size, and *dilation* is the dilated convolution. In the dilation, the stride is 1, the padding size is 6, and the convolutional kernel size is 6 with 3×3 parameters.

#### 3.1.3. Anchor Generation

**Figure 3:**
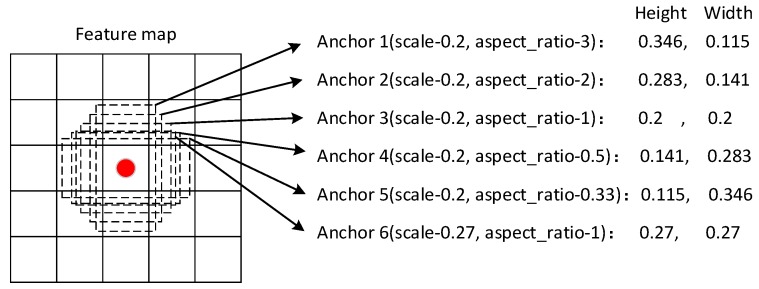
Anchor generation flow. For each pixel on the feature map, six anchor shapes are generated that share the same center with the scales of 0.2 and 0.27. In addition, the center of each anchor is the pixel center. In the figure, the center is (0.5, 0.5).

The anchor in the proposed network plays two roles. The first role is in the data selection procedure. The IOU of the ground truth and an anchor decides whether this anchor is a positive sample [31]. Traditionally, if the IOU is more than 50%, the anchor is a positive sample. The second role is in the training and testing procedure. In the training procedure, the classification and location loss are computed by the anchors. In the testing procedure, the bounding box results are computed by the anchor location layers and, after the non-maximum suppression of the bounding box results, the detection results are computed. Figure 3 shows the flow of the anchor generation.

Traditional object detection methods suggest processing an image at different scales and then combines the results. Since we have the multiscale feature representation, we can utilize these feature maps with different scales in a single network to have a similar effect with the traditional image processing method.

Similar to the Faster RCNN and the SSD, the anchors are generated on the feature maps by dense sampling. First, to handle different sizes of objects, different scales of objects are detected on different feature maps. Therefore, in different feature maps, we allocate different scales. In the feature map with low resolution, the anchor scale is large, and in the feature map with high resolution, the anchor scale is small. Second, for a certain scale, the anchor should have different aspect ratios. The design of these aspect ratios are decided by the object’s aspect ratios. Suppose that we have m feature maps and the detection is applied to these m feature maps, the anchor scale of the feature map is computed as follows:

(1) $$s_{n}=s_{\min}+\frac{s_{max}-s_{\min}}{m-1}(n-1),\;n\in [1,m]$$

In the formulation, $s_{\min}$ is the minimum scale of the objects to be detected, and $s_{max}$ is the maximum scale of the objects to be detected. *n* represents the *n*th square feature map. $s_{n}$ is the scale of the certain feature map. According to the scales, the anchor area is obtained as $anchor_{area}$ = $s_{n}^{2}$.

For the aspects of the anchors, different aspect ratios are set according to the object’s aspect ratios. Traditionally, the aspect ratios contain $a_{r}$ = [1, 2, 3, $\frac{1}{2}$, $\frac{1}{3}$]. Furthermore, for the scale consistency, a scale of $s_{n}^{\prime }=\sqrt{s_{n}s_{n+1}}$ and the aspect ratio of 1 are also considered. The width and height are computed as follows:

(2) $$\left\{ \begin{array}{l} anchor\_w_{n}=s_{n}\sqrt{a_{r}}\\ anchor\_h_{n}=s_{n}/\sqrt{a_{r}} \end{array}\right.$$

Finally, there are six anchors for each pixel in the feature maps.

In the proposed model, we allocate the different scales from 0.1 to 0.95, in which 0.95 indicates that the object occupies the entire image and 0.1 indicate the down-sampling rate on Conv4_3. Moreover, we set the starting scale of Conv6_2 is 0.2. The aspect ratios contain $a_{r}$ = [1, 2, 3, $\frac{1}{2}$, $\frac{1}{3}$] and aspect ratio of 1 for scale of $\sqrt{s_{n}s_{n+1}}$.

Table 2 shows the anchor details with the height, weight and number of each feature map in Section 3.1.2. In the table, for clarity, the channel number of the feature map is hidden. To include more objects with different aspect ratios and scales, there are six anchor shapes for each pixel on the feature map.

#### 3.1.4. Anchor Prediction

**Figure 4:**
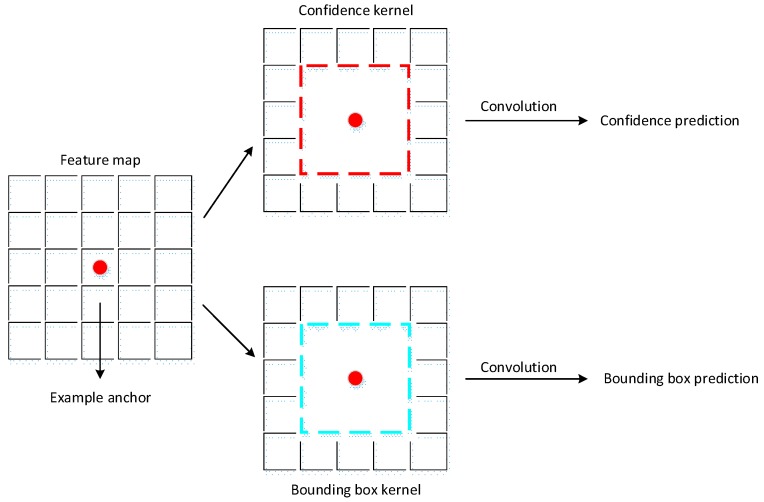
Confidence and bounding box prediction from an anchor. The red point is the center of the example anchor. There are two kernels of 3×3 for the confidence prediction and the bounding box prediction for this anchor. The red kernel is the confidence kernel and the blue kernel is the bounding box prediction kernel.

After associating a set of anchors with each feature map, the anchor prediction is the next key procedure during the algorithm. At each anchor, we predict the offsets relative to the anchor shapes and the per-class scores that indicate the presence of a class instance in this anchor. Suppose that the class number that we want to predict is *n*. For each anchor, the output size is (*n* + 1) + 4. (*n* + 1) is the class number and background, and 4 is the bounding box offset for this anchor. Figure 4 shows the prediction procedure of an anchor.

Referring to the multiscale feature representation in Section 3.1.2, Table 3 shows the detailed prediction kernels. The confidence output of each anchor is (n+1)×6 dimensions with (n+1) classes and six shapes. The bounding box output of each anchor is (n+1)×4 dimensions with (n+1) classes and (*bx*, *by*, *bw*, *bh*).

#### 3.1.5. Training Sample Selection

In the training procedure, the training samples are generated from the anchors. A matching strategy between the anchors and the ground truths is applied. The strategy begins by matching each anchor to the ground truth. Different from Multibox, the positive samples are the anchors with a Jaccard overlap higher than a threshold. The others are the negative samples. This strategy simplifies the training sample selection problems and provides the network with more positive samples.

Moreover, to improve performance, the network also incorporates hard example mining and data augmentation.

1. Hard Example Mining

Since the negative samples are selected from the background, the number of negative samples is much larger than the number of positive samples. This difference causes a significant imbalance between the positive and negative training samples. To overcome this difficulty, following OHEM [32], we sort the samples by their prediction scores. By selecting the top scores, we restrict the ratio between the negative and positive training samples to 3:1. In this way, we can avoid training mainly on the negative samples.

2. Data Augmentation

To improve the generalization of the network, we also apply data augmentation. Similar to SSD, each training image is augmented by one of the following options:

- Use the entire original input image;
- Sample a patch so that the minimum Jaccard overlap with the objects is 0.1, 0.3, 0.5, 0.7, or 0.9;
- Randomly sample a patch.

After the aforementioned sampling step, each augmented image is resized to a fixed size and is horizontally flipped with the probability of 0.5.

### 3.2. Loss Function

In the proposed network, the loss function mainly contains two parts: the detection loss and the correlation loss. The detection loss relates to the object classification and the object bounding box regression. The correlation loss is the neighboring object feature correlation, which could involve the time-series information of the network.

#### 3.2.1. Detection Loss

Detection loss relates to an object in a single frame, and it involves the frame information concerning the object. Similar to the Faster RCNN [6], detection loss consists of a combined classification loss $L_{cls}$ and a bounding box regression loss $L_{bbox}$. The overall detection loss is the sum of $L_{cls}$ and $L_{bbox}$. Suppose that *f* is a feature in a certain anchor with a Jaccard overlap that is higher than 0.5. Then:

(3) $$L_{\det}(f,l,g,c)=\frac{1}{n}(aL_{bbox}(f,l,g)+L_{cls}(f,c)),$$

where *n* is the number of anchors whose Jaccard overlap is higher than 0.5; *f* is the feature of the certain anchor; *c* is the classification score of the anchor; and *l* and *g* are the bounding box offsets and ground truth, respectively.

Similar to SSD, $L_{cls}$ is formed by multi class softmax:

(4) $$L_{cls}(f,c)=-(\sum_{i\in positive}^{n}f_{ij}^{label}\log({\overset{⌢}{c}}_{i}^{label})+\sum_{i\in negtive}^{n}\log({\overset{⌢}{c}}_{i}^{label})),$$

where:

(5) $${\overset{⌢}{c}}_{i}^{label}=\frac{\exp(c_{i}^{label})}{\sum_{label}c_{i}^{label}},$$

In the formulation, $c_{i}^{label}$ is the anchor classification prediction score of the certain *label* in the *i*th anchor box. For example, $c_{5}^{person}$ is the prediction score of *person* in the 5th anchor box. Let $f_{ij}^{label}=\left[1,\;0\right]$ be the indicator for the *i*th anchor box to the *j*th ground truth of *label*. Moreover, the background *label* is 0. Therefore, if i∈negtive, then $c_{i}^{label}$ = $c_{i}^{0}$.

$L_{bbox}$ is based on the Huber loss [33] between the ground truth and the bounding box. The Huber loss is less sensitive to outliers in the data than the squared error loss. $L_{bbox}$ is formulated as follows:

(6) $$L_{loc}=\sum_{i\in positive}^{n}\sum_{k\in (bx,by,bw,bh)}f_{ij}^{label}Huber(pre_{i}^{m}-{\overset{⌢}{g}}_{j}^{m}),$$

in which:

(7) $$Huber(x)=\left\{ \begin{array}{ll} 0.5x^{2} & \left(\left|x\right|\leq \alpha \right)\\ \alpha (\left|x\right|-0.5\alpha ) & \left(\left|x\right|>\alpha \right) \end{array}\right.$$

Similar to R-CNN, α is a hyper-parameter and set to 1.

Suppose that the bounding box has four parameters, which are (*bx*, *by*, *bw*, *bh*). (*bx*, *by*) is the center of the bounding box; and (*bw*, *bh*) is the width and height, respectively. *Pre* and *g* refer to the prediction box and the ground truth box, respectively. *i* and *j* are as the same as $L_{cls}$.

Moreover, when formulating $L_{bbox}$, we adopt the parameterizations of the anchor bounding box(*a*) and the ground truth box(*g*) following R-CNN [8]:

(8) $$\left\{ \begin{array}{l} {\overset{⌢}{g}}_{j}^{bx}=\frac{g_{j}^{bx}-a_{i}^{bx}}{a_{i}^{bw}}\\ {\overset{⌢}{g}}_{j}^{by}=\frac{g_{j}^{by}-a_{i}^{by}}{a_{i}^{bh}}\\ {\overset{⌢}{g}}_{j}^{bw}=\log(\frac{g_{j}^{bw}}{a_{i}^{bw}})\\ {\overset{⌢}{g}}_{j}^{bh}=\log(\frac{g_{j}^{bh}}{a_{i}^{bh}}) \end{array}\right.$$

This parameterization enhances the effect of the center (*bx*, *by*) and weakens the effect of the width (*bw*) and height (*bh*).

#### 3.2.2. Correlation Loss

Detection loss is mainly concerned with intra-frame information. However, in video sources, detection loss does not consider inter-frame information. Correlation loss is a strategy that involves the inter-frame information by measuring the feature difference between frames.

As is known, the traditional detection frameworks based on deep neural networks are discriminative algorithms. The main concern is how to plot a line or surface in the feature space. If the feature is on the left side of the line, the feature is generated by the positive sample. According to the well-constructed feature space of a convolutional neural network, the linear classifier (Softmax) could easily differentiate the negative features from the positive features. Due to the discriminative algorithm characteristics, the feature in the feature space is scattered. In video object detection, the object is continuously moving. Therefore, when the training dataset is completed, the intra-frame-based detection framework may have a favorable result.

The proposed correlation loss is inspired by the tracking task. In the tracking task [16,34,35,36,37], the feature consistency is the key point to judge the tracking result, especially in the correlation-based tracking algorithm such as the correlation filter [34]. In the deep feature [12] and flow-guided feature [38], according to the object’s movement, the feature is copied or aggregated from the key frames of other frames. In this way, the feature stability has been guaranteed. Different from these approaches, we formulate a correlation loss to supervise the feature consistency.

As shown in Figure 1, the correlation loss is computed by the Siamese network, which is a two-path network that uses replication. since we construct the multiscale feature map, the correlation loss is generated from it. The correlation loss $\left(L_{corr}\right)$ is the combination of the center value loss $\left(L_{center\_value}\right)$ and the anchor coordinate loss ($L_{coordinate}$):

(9) $$L_{corr}(f^{t},f^{t+1},map^{t+1},a^{t+1})=\frac{1}{n}(L_{center\_value}(f^{t},f^{t+1})+L_{coordinate}(f^{t},map^{t+1},a^{t+1})),$$

where *n* is the number of ground truths; $f^{t}$ and $f^{t+1}$ are the anchor center features of the neighboring frame *t* and *t* + 1, respectively; and $a^{t+1}$ is the positive anchor box in frame *t* + 1. Each positive anchor box has 4 parameters (*bx*, *by*, *bw*, *bh*). $map^{t+1}$ is the entire scale feature map in frame *t* + 1. Moreover, the positive anchor is different from the positive sample selection. In the correlation loss procedure, the anchor selection is the maximum Jaccard overlap between the ground truth box and the anchors, which means that the number of positive anchors is the same as the ground truth.

First, the correlation loss is a value that compares the correlation distance between the same labelled object features in the neighboring frame.

The following is the formulation of $\left(L_{center\_value}\right)$:

(10) $$\begin{array}{l} L_{center\_value}(f^{t},f^{t+1})=\sum_{i\in positive}^{n}f_{i}^{t,label\_t,track\_t}⊗f_{j}^{t+1,label\_t+1,track\_t+1},\\ if\;(label\_t=label\_t+1)\;and\;(track\_t=track\_t+1) \end{array}$$

Here, ⊗ is the correlation operation. The correlation operation measures the consistency of the feature. *label_t* and *track_t* are the ground truth label and the track ID, respectively, that relate to this anchor. By searching all the positive anchors in the neighboring frame, we can obtain the one-to-one corresponding positive anchor with its label and track ID in frame *t* and frame *t* + 1. $f^{t,label\_t,track\_t}$ is the center value of the positive anchor.

**Figure 5:**
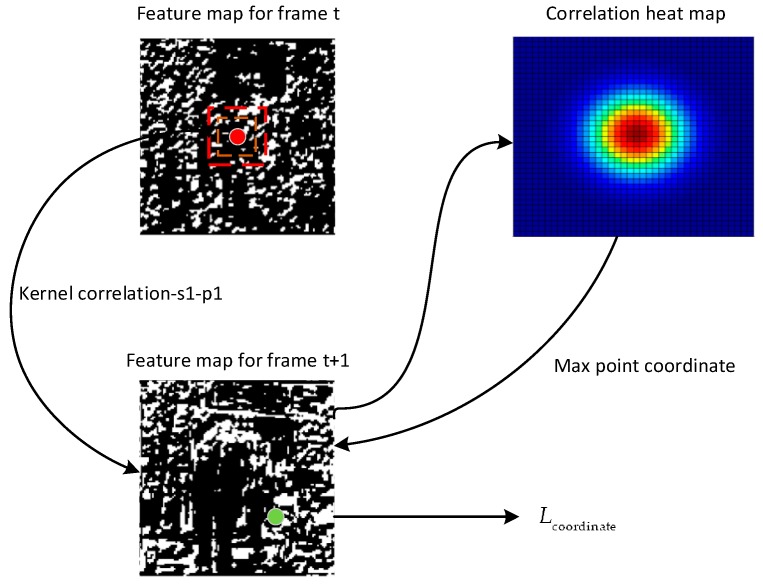
Coordinate loss computation flow. The red and green points are the positive anchors of frame *t* and frame *t* + 1, respectively. A score heat map is computed with the correlation operation by using a 3×3 kernel in frame *t* with the red point center and the feature map of frame *t* + 1, similar to tracking on frame *t* + 1. After obtaining the max point coordinate, $L_{coordinate}$ can be computed by the max point coordinate and the green point coordinate.

Second, the anchor coordinate loss measures the tracking result loss on the neighboring frame feature. Inspired by the correlation filter, we take the positive anchor in frame *t* as the first-frame tracking ground truth. We obtain the response map in frame *t* + 1. Then, the coordinate of the highest response is the tracking result in frame *t* + 1. Figure 5 shows the $L_{coordinate}$ computation flow.

Then, the anchor coordinate loss is formed as:

(11) $$\begin{array}{l} L_{coordinate}(f^{t},map^{t+1},a^{t+1})=\sum_{i\in positive}^{n}Dis(T(f_{i}^{t,label\_t,track\_t},map^{t+1}),a^{t+1,label\_t+1,track\_t+1})/map\_size,\\ if\;(label\_t=label\_t+1)\;and\;(track\_t=track\_t+1) \end{array}$$

where:

(12) $$T(f_{i}^{t,label\_t,track\_t},map^{t+1})=max({\overset{⌢}{f}}_{i}^{t,label\_t,track\_t}⊗map^{t+1})[bx,by]$$

The *T* (*Track*) formulation obtains the center (*bx*, *by*) of the max response location. In $L_{coordinate}$, *Dis* computes the center Euclidean distance of the tracking result and the positive anchor in frame *t* + 1. Moreover, in case the coordinate loss is high, we normalize it by the size of the feature map. By applying the correlation loss, the object feature in the neighboring frame can be more consistent.

## 4. Experiment and Results

We report the results of the ImageNet VID validation dataset. The training set contains the ImageNet VID training dataset. The performance of our method is compared to the R-CNN [8], Fast R-CNN [5], original SSD [10], T-CNN [15], and TPN + LSTM [39], and the winner of the competition of ImageNet VID 2015 [13]. The detailed evaluation metrics are described in Section 4.1. All the methods in the experiments were programmed based on the Pytorch deep learning framework. The computational resources include a TITAN X GPU, 128 GB of memory, and an Intel Xeon E5-2670 CPU (2.30 GHz). The operating system used is Ubuntu 14.04. Moreover, in order to show the effectiveness of the proposed method, we also evaluate our model on the YouTube Object (YTO) dataset and the Unsupervised [40], YOLO [22], Context [41], a_LSTM [42], T-CNN [15], Base [10] models are selected for comparison.

### 4.1. ImageNet Dataset

**Figure 6:**
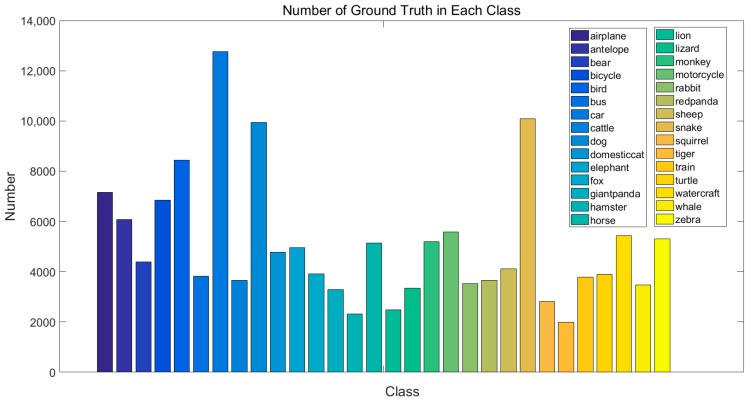
The number of ground truths in each class.

We evaluate our method by using the 2015 ImageNet object detection from a video (VID) [23] dataset that contains 30 classes in 3862 training and 555 validation videos. The 30 object categories in ImageNet VID are a subset of the 200 categories in the ImageNet DET dataset. The objects have ground truth annotations for their bounding box and a tracking ID in a video. Since the ground truth for the test set is not publicly available, we measure the performance as the mean average precision (mAP) over 30 classes on the validation set by following the protocols in [12,13,15,38,39], which is standard practice. Figure 6 shows the number of ground truths in each class, in which we can see that the training ground truth in each class is unbalanced. Then, we subsample the VID training set by using only 30 frames from each video.

**Figure 7:**
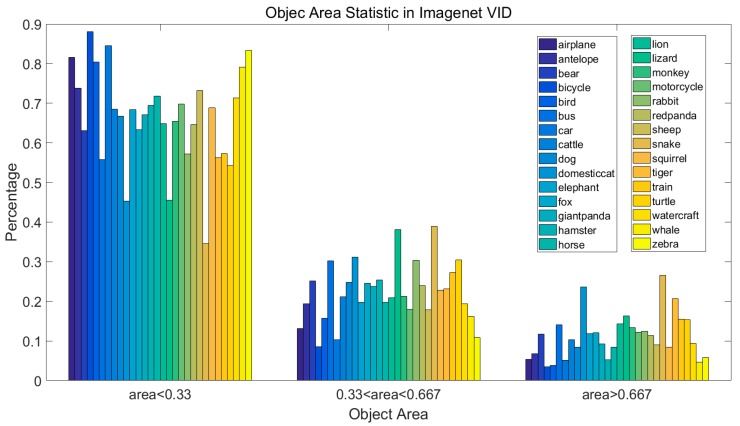
The object area characteristics of the Imagenet VID dataset.

Figure 7 shows the object area statistical information in the dataset, in which we can see more than half of the objects have areas lower than 0.33.

**Figure 8:**
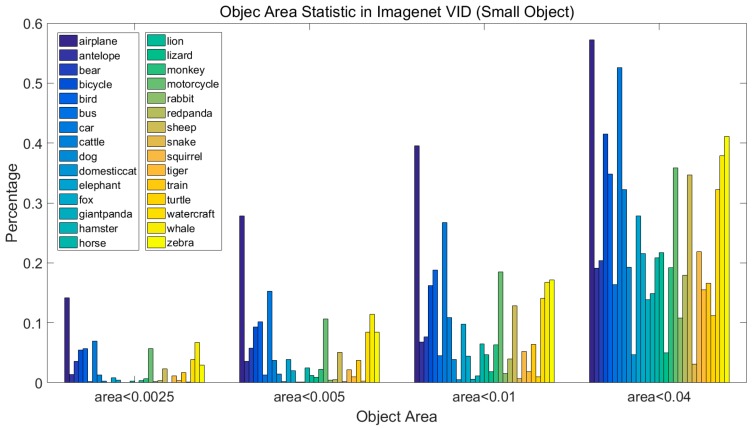
The small object area characteristics of the Imagenet VID dataset.

The positive samples are anchors with Jaccard overlap of ground truths of more than 0.5. If the Jaccard overlaps between all of the anchors and ground truths are lower than 0.5, there would be no positive samples. Figure 8 shows the statistical information of small objects. In this dataset, if the scale of the smallest anchor is 0.1, the framework will miss about 10% of the ground truths.

### 4.2. Model Training

Baseline-single shot multibox: The baseline is SSD300, which means the input is resized to 300×300. The multiscale feature map sizes are 38×38, 19×19, 10×10, 5×5, 3×3, and 1×1. There are six anchor shapes in the multiscale feature map, which contains [1, 2, 3, $\frac{1}{2}$, $\frac{1}{3}$] with an aspect ratio of 1, for a scale of $\sqrt{s_{n}s_{n+1}}$. The backbone is the VGG 16 net with the fully-connected layers removed. Following SSD300, we change pool5 from 2×2−s2 to 3×3−s1 and use an à trous algorithm to fill the “holes”.

The baseline uses conv4_3, conv6_2, conv7_2, conv8_2, conv9_2, and conv10_2 to predict both the locations and confidences. The baseline sets the anchor with a scale of 0.1 on conv4_3, and the other layers are initialized by the “Xavier” method [43]. For all prediction layers, we included six anchor shapes as described in Section 3.1.3. The baseline uses the $10^{-3}$ learning rate for 200,000 iterations. The training was continued for 200,000 iterations with $10^{-4}$ and $10^{-5}$. Moreover, the optimization trick has a momentum of 0.9 and a weight decay of 0.0005.

Proposed network: Our network is established on SSD300, whose input image size is also resized to 300×300. The training differences between the proposed network and the baseline are as follows:

1. Input image

The input image is a pair of neighboring frames (frame *t* and frame *t* + 1). The neighboring frames must have at least one object pair with the same label and track ID. If one of the neighboring frames is empty, this frame pair is removed.

2. Correlation loss computation

Correlation loss $\left(L_{corr}\right)$ has two subsets: $\left(L_{center\_value}\right)$ and ($L_{coordinate}$). $\left(L_{center\_value}\right)$ is computed by the center values of the positive anchors. In $L_{coordinate}$, when computing the response map, the kernel size is 3×3, and its center is the positive anchor center. Furthermore, frame *t* + 1 is padded by one pixel.

3. Learning rate

Since the loss in the proposed network is more than the baseline, we first warm up the network with a learning rate of $10^{-4}$ for 10,000 iterations. Then, the proposed network uses the $10^{-3}$ learning rate for 200,000 iterations. The training was continued for 200,000 iterations with $10^{-4}$ and $10^{-5}$.

4. Training time

The training time of the baseline is almost three days. Due to the correlation loss computation and the detection loss that contains two images, the training time of our method is longer than the baseline. Following the above training process, the entire training time is approximately seven days.

In addition to the above procedure, the proposed network training is mostly the same as the baseline. The proposed network also uses six layers to predict, and each layer’s anchor shape is six. The optimization trick that we use is a momentum of 0.9 and a 0.0005 weight decay.

### 4.3. Testing and Results

We test the baseline and the proposed network on the validation dataset with subsampling. Moreover, we also adopt Soft-NMS [19] to accurately fix the candidate bounding boxes. Moreover, the Seq-NMS [14], which is a post-processing method, is applied after the detection process. Table 4 shows the mAP results on the validation dataset. From the table, the baseline SSD300 achieves a mAP of 67.9%. Our proposed method achieves 69.5%, which is a 1.6% improvement compared to the baseline.

From Table 4, we compare our proposed network with R-CNN [8], Fast R-CNN [5], T-CNN [15], TPN + LSTM [39], the baseline and the winner in the competition of ImageNet VID 2015 [13] (multi-model). The R-CNN and Fast R-CNN are baselines designed for still images. The last list is the winner in the competition of ImageNet VID 2015 and the result is the fusion of DeepID net, CRAFT and post-processing procedures. For the single model, the proposed network achieves the best score, which is a 1.1% improvement over TPN + LSTM.

Moreover, because our proposed network is based on SSD300, and because the testing process is similar, the test time per image is similar. Based on our equipment (Titan X), the testing time per image is approximately 32 fps because there are more anchors than the original SSD.

**Figure 9:**
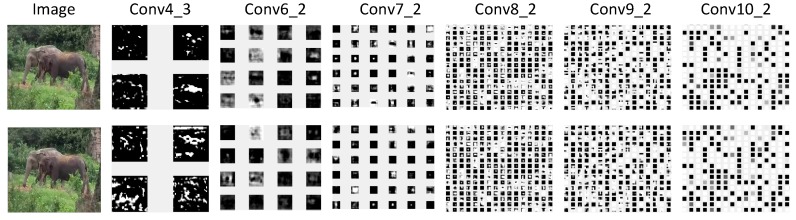
Multiscale feature representation of the network. The first line is the multiscale feature representation of the proposed method and the second line is the feature of the baseline.

During the network feed-forward, Figure 9 shows some middle features, containing conv4_3, conv6_2, conv7_2, conv8_2, conv9_2, and conv10_2.

To measure the effect on features, whether involving the correlation loss or not, we extract features of the anchor with the maximum Jaccard overlap of ground truth. Then, computing the similarity of the neighbouring frame, the similarity metric is the Euclidean distance. For each class, we choose 10 pairs of neighbouring frames and the similarity metric is the average of those similarities. In order to avoid the effect of the channel size, the similarity index is normalized by channels:

(13) $$Similarity=\sum_{a}^{n}\sqrt{{\left(f_{frame\_t}^{a}-f_{frame\_t+1}^{a}\right)}^{2}}/channels/n$$

Here, $f_{frame\_t}^{a}$ and $f_{frame_{t}+1}^{a}$ are the features of the certain anchor on the neighbouring frame. The *channel* is the number of the feature map.

**Figure 10:**
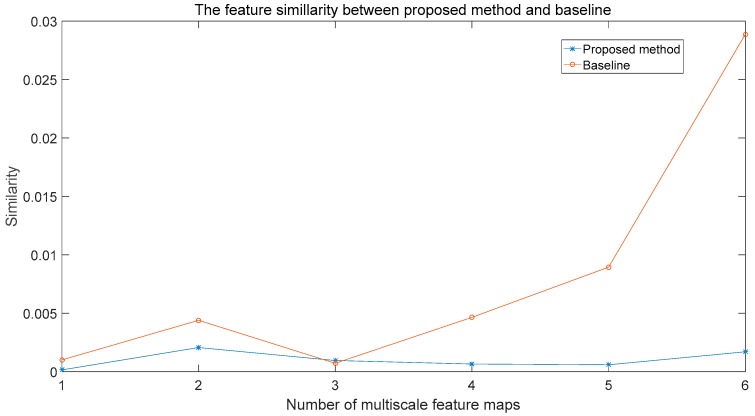
The feature similarity of the proposed method. The horizontal axis is the number of the multiscale feature map and the vertical axis is the similarity index.

Figure 10 shows the feature’s similarity of the proposed network and baseline. We can see our proposed method maintains a better feature similarity than the baseline.

**Figure 11:**
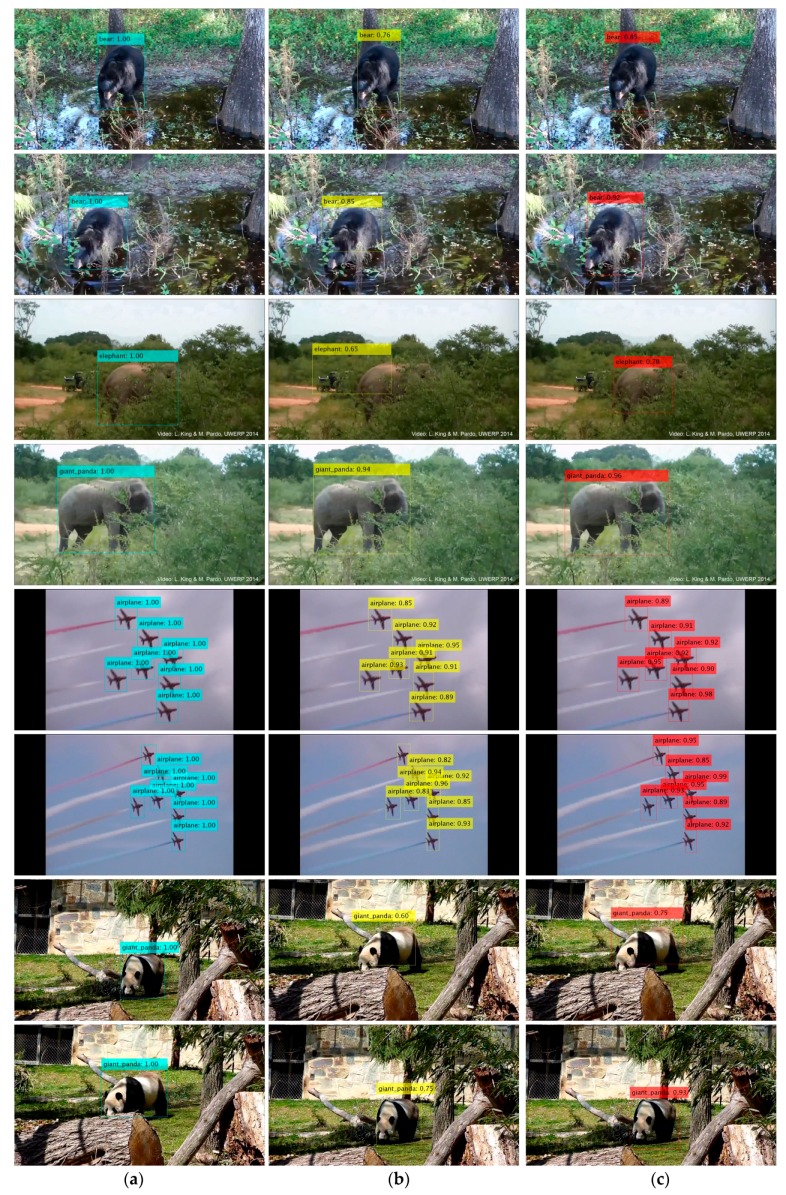
Results of the validation dataset. (**a**) Blue bounding boxes are the ground truths. (**b**) Yellow bounding boxes are the baseline results. (**c**) Red bounding boxes are our results.

Figure 11 shows the detection results of the validation dataset.

### 4.4. Model Analysis

The number of anchor shapes and multi-scale feature maps are the key hyper-parameters in the proposed framework. To evaluate the effects of different number of anchor shapes and feature maps, additional comparison experiments are conducted on the ImageNet VID dataset.

#### 4.4.1. Number of Anchor Shapes

In the proposed framework, the number of anchor shapes for each pixel in the multi-scale feature map is one of the key hyper-parameter and the number of anchor shapes could affect the detection performance and speed. To analyze how the number of anchors affect the detection performance and speed, we conduct a comparison experiment between different numbers of anchors. Table 5 shows the details about the effect on detection performance and speed. The settings of this experiment are as following:

1. Anchor-6: For each pixel in multi-scale feature map, there are six anchor shapes in the multiscale feature map, which contains [1, 2, 3, $\frac{1}{2}$, $\frac{1}{3}$] and an aspect ratio of 1 for a scale of $\sqrt{s_{n}s_{n+1}}$. In our framework, the total number of anchors is 11,640.
2. Anchor-4: For each pixel in multi-scale feature map, there are four anchor shapes in the multiscale feature map, which contains [1, 2, $\frac{1}{2}$] and an aspect ratio of 1 for a scale of $\sqrt{s_{n}s_{n+1}}$. In our framework, the total number of anchors is 7760.
3. Anchor-4 and 6: In this setting, we follow the original SSD setting. In Conv_4_3, Conv9_2, and Conv10_2, there are four anchor shapes in the multiscale feature map, which contains [1, 2, $\frac{1}{2}$] and an aspect ratio of 1 for a scale of $\sqrt{s_{n}s_{n+1}}$. Then, in Conv_6_2, Conv7_2, and Conv8_2, there are six anchor shapes in the multiscale feature map, which contains [1, 2, 3, $\frac{1}{2}$, $\frac{1}{3}$] and an aspect ratio of 1 for a scale of $\sqrt{s_{n}s_{n+1}}$. In our framework, the total number of anchors is 8732.

From the table, we can see that more anchors refer to better mean average precision. Additionally, more anchors need more computational resources and the detection speed is lower. If we remove the aspect ratio of 3 and $\frac{1}{3}$, the performance drops 1.6% and the detection speed has an 18 fps bonus.

#### 4.4.2. Number of Multi-Scale Feature Maps

In our framework, the number of multi-scale feature maps is also a key hyper-parameter. To investigate the effect of the multi-scale feature representation, we do another comparison experiment between different numbers of multi-scale feature maps. Table 6 shows the details about the effect on detection performance and speed. Moreover, the anchor shapes in each pixel is six, which consists of [1, 2, 3, $\frac{1}{2}$, $\frac{1}{3}$] and an aspect ratio of 1 for a scale of $\sqrt{s_{n}s_{n+1}}$. The followings are the experimental settings:

1. Feature-6: In this setting, we use six feature maps, which are Conv4_3, Conv6_2, Conv7_2, Conv8_2, Conv9_2, and Conv10_2. In our framework, the total number of anchors is 11,640.
2. Feature-5: In this setting, we use five feature maps, which are Conv4_3, Conv6_2, Conv7_2, Conv8_2, and Conv9_2. In our framework, the total number of anchors is 11,634.
3. Feature-4: In this setting, we use four feature maps, which are Conv4_3, Conv6_2, Conv7_2, and Conv8_2. In our framework, the total number of anchors is 11,580.
4. Feature-3: In this setting, we use three feature maps, which are Conv4_3, Conv6_2, and Conv7_2. In our framework, the total number of anchors is 11,430.

From the table, we can see that more feature maps can significantly enhance the mean average precision. Since the anchors are mostly generated on feature maps with lower resolution, the speed between the settings does not make a great difference. Compared with Feature-3, Feature-6 has a 2.7% bonus on mean average precision.

### 4.5. Evaluation of the YouTube Object (YTO) Dataset

In order to show the effectiveness of the proposed network, we evaluate our model on a video object detection task with the YTO dataset [44].

#### 4.5.1. YouTube Object Dataset

The YTO dataset contains 10 object classes, which include an airplane, bird, boat, car, cat, cow, dog, horse, motor-bike, and train. Moreover, these 10 object classes are a subset of the ImageNet VID dataset and these objects are also moving objects. Different from the VID dataset which contains full annotations on all video frames, the YTO training dataset is weakly annotated, i.e., each video is only ensured to contain one object of corresponding class, and only a few frames, whereas the objects in the YTO test dataset are all annotated. In total, the YTO dataset contains 155 videos. However, it only contains 6087 annotated frames; among them 4306 are for training and 1781 are for testing. The weak annotation makes it infeasible to train the proposed network on the YTO dataset.

Following [13,15], since the YTO classes are a subset of the VID dataset classes, we only use this dataset for evaluation and can directly apply the trained models on the YTO dataset for evaluation. The evaluation metric is the same as the ImageNet VID dataset.

#### 4.5.2. Evaluation Results

We evaluate the model trained on ImageNet to the YTO test dataset and several state-of-the-art methods are selected for comparison. Table 7 shows the detailed AP lists computed by Unsupervised [40], YOLO [22], Context [41], a_LSTM [42], T-CNN [15], Base [10] and our own YTO test dataset.

From the table, we can see that our proposed framework outperforms by a large margin. Compared with the baseline, our proposed method has around a 4.4% improvement and, compared with T-CNN, our proposed method has a 4.0% bonus.

## 5. Conclusions

In this paper, we propose a fast and accurate object detection network for video sources. The proposed network is a single-shot object detection network. Unlike the traditional single-frame-based object detection network, our proposed network involves frame-to-frame information by using the object pair relations among neighboring frames. Following the Siamese network, we formulate a correlation loss to restrain the deep features. In this way, we incorporate correlation into the discriminative algorithm. The backbone network is the VGG16 model that reduced the fully-connected layers. Based on the backbone network, we establish a multiscale feature representation to predict detections on multiple layers. Different anchor scales are applied to different feature maps for different object scales. Hard example mining and data augmentation are also used to balance the training samples and to test the generalizations. Our proposed model has been tested on a large object detection dataset, namely, the ImageNet VID dataset. This dataset is the largest video object detection dataset. Compared to the baseline, our proposed network has a 1.6% bonus, and the test time does not increase. In order to show the effectiveness of the proposed framework, we evaluate the model on YTO dataset and the proposed framework has a 4.4% bonus compared to the baseline.

Although our proposed network has a better performance than the baseline, it still has some limitations. The first limitation is the use of time-series information. The correlation loss is rigid to the feature map. The second limitation is that we do not fully use the tracking information. The tracking process is only on the feature map and does not output the exact tracking result. If the network could output the exact tracking result, the testing time could be faster.

## Figures and Tables

**Table 1:** Details of the multiscale feature representation.

Stage	Conv Kernel Size	Feature Map Size	Usage	Options
Conv4_3	(3×3×512)×512	38×38×512	Detection for scale 1	*s*-1, *p*-1
Conv6_1	(3×3×512)×1024	19×19×1024	Enlarge receptive field	*dilation*
Conv6_2	(1×1×1024)×1024	19×19×1024	Detection for scale 2	*s*-1, *p*-0
Conv7_1	(1×1×1024)×256	19×19×256	Reduce channels	*s*-1, *p*-0
Conv7_2	(3×3×256)×512	10×10×512	Detection for scale 3	*s*-2, *p*-1
Conv8_1	(1×1×512)×128	10×10×128	Reduce channels	*s*-1, *p*-0
Conv8_2	(3×3×128)×256	5×5×256	Detection for scale 4	*s*-2, *p*-1
Conv9_1	(1×1×256)×128	5×5×128	Reduce channels	*s*-1, *p*-0
Conv9_2	(3×3×128)×256	3×3×256	Detection for scale 5	*s*-1, *p*-0
Conv10_1	(1×1×256)×128	3×3×128	Reduce channels	*s*-1, *p*-0
Conv10_2	(3×3×128)×256	1×1×256	Detection for scale 6	*s*-1, *p*-0

**Table 2:** Anchor details in multiscale feature representation.

Feature	Feature Map Size	Anchor Height	Anchor Width	Number
Conv4_3	38×38	0.0707, 0.0577, 0.1414 0.1732, 0.1000, 0.1414	0.1414, 0.1732, 0.0707 0.0577, 0.1000, 0.1414	8864
Conv6_2	19×19	0.1414, 0.1155, 0.2828 0.3464, 0.2000, 0.2784	0.2828, 0.3464, 0.1414 0.1155, 0.2000, 0.2784	2166
Conv7_2	10×10	0.2740, 0.2237, 0.5480 0.6712, 0.3875, 0.4720	0.5480, 0.6712, 0.2740 0.2237, 0.3875, 0.4720	600
Conv8_2	5×5	0.4066, 0.3320, 0.8132 0.9959, 0.5750, 0.6621	0.8132, 0.9959, 0.4066 0.3320, 0.5750, 0.6621	150
Conv9_2	3×3	0.5392, 0.4402, 1.0783 1.3207, 0.7625, 0.8511	1.0783, 1.3207, 0.5392 0.4402, 0.7625, 0.8511	54
Conv10_2	1×1	0.6718, 0.5485, 1.3435 1.6454, 0.9500, 1.0395	1.3435, 1.6454, 0.6718 0.5485, 0.9500, 1.0395	6

**Table 3:** Details of the prediction kernel.

Feature	Feature Map Size	Confidence Kernel	Location Kernel
Conv4_3	38×38×512	3×3×512×(n+1)×6	3×3×512×(n+1)×4
Conv6_2	19×19×1024	3×3×1024×(n+1)×6	3×3×1024×(n+1)×4
Conv7_2	10×10×512	3×3×512×(n+1)×6	3×3×512×(n+1)×4
Conv8_2	5×5×256	3×3×256×(n+1)×6	3×3×256×(n+1)×4
Conv9_2	3×3×256	3×3×256×(n+1)×6	3×3×256×(n+1)×4
Conv10_2	1×1×256	3×3×256×(n+1)×6	3×3×256×(n+1)×4

**Table 4:** Average precision of each class and mAP. The bold values in the table are the best results among single models for a certain class.

Class	[8]	[5]	[15]	[39]	Baseline	Our	[13]
Airplane	64.5	82.1	72.7	**84.6**	79.3	81.2	83.7
Antelope	71.4	78.4	75.5	**78.1**	73.2	73.5	85.7
Bear	42.6	66.5	42.2	**72.0**	65.0	70.2	84.4
Bicycle	36.4	65.6	39.5	67.2	**72.5**	72.3	74.5
Bird	18.8	66.1	25	68	70.9	**71.5**	73.8
Bus	62.4	77.2	64.1	**80.1**	76.8	78.6	75.7
Car	37.3	52.3	36.3	**54.7**	49.2	50.1	57.1
Cattle	47.6	49.1	51.1	61.2	63.8	**65.3**	58.7
Dog	15.6	57.1	24.4	**61.6**	56.6	60.4	72.3
Dc_cat	49.5	72.0	48.6	**78.9**	72.6	70.1	69.2
Elephant	66.9	68.1	65.6	71.6	78.9	**82.6**	80.2
Fox	66.3	76.8	73.9	83.2	85.6	**85.9**	83.4
Giant_panda	58.2	71.8	61.7	78.1	79.8	**81.2**	80.5
Hamster	74.1	89.7	82.4	**91.5**	86.5	87.5	93.1
Horse	25.5	65.1	30.8	66.8	73.5	**75.2**	84.2
Lion	29	20.1	34.4	21.6	46.5	**47.8**	67.8
Lizard	68.7	63.8	54.2	**74.4**	69.4	71.5	80.3
Monkey	1.9	34.7	1.6	36.6	**52.6**	50.3	54.8
Motorcycle	50.8	74.1	61.0	**76.3**	70.8	72.5	80.6
Rabbit	34.2	45.7	36.6	51.4	59.1	**61.8**	63.7
Red_panda	29.4	55.8	19.7	70.6	67.8	**71.9**	85.7
Sheep	59.0	54.1	55.0	**64.2**	38.7	40.0	60.5
Snake	43.7	57.2	38.9	61.2	59.2	**62.3**	72.9
Squirrel	1.8	29.8	2.6	42.3	83.4	**85.1**	52.7
Tiger	33.0	81.5	42.8	**84.8**	76.8	78.1	89.7
Train	56.6	72.0	54.6	**78.1**	69.3	71.2	81.3
Turtle	66.1	74.4	66.1	**77.2**	72.9	74.5	73.7
Watercraft	61.1	55.7	**69.2**	61.5	63.4	65.6	69.5
Whale	24.1	43.2	26.5	**66.9**	46.8	51.8	33.5
Zebra	64.2	89.4	68.6	**88.5**	74.9	75.2	90.2
mAP	45.3	63.0	47.5	68.4	67.9	**69.5**	73.8

**Table 5:** Detection performance on different number of anchor shapes. The bold values in the table are the fastest detection speed and best performance.

Settings	Anchor-6	Anchor-4	Anchor-4 and 6
Anchor number	11,640	7760	8732
Detection speed	32 fps	**51 fps**	46 fps
Mean AP	**69.5**	67.9	68.3

**Table 6:** Detection performance on different number of feature maps. The bold values in the table are the fastest detection speed and the best performance.

Settings	Feature-6	Feature-5	Feature-4	Feature-3
Anchor number	11,640	11,634	11,580	11,430
Detection speed	32 fps	32 fps	32 fps	**33 fps**
Mean AP	**69.5**	69.3	69.1	66.8

**Table 7:** Detection performance on the YTO dataset. The bold values in the table are the best results among single models for a certain class.

Class	[40]	[22]	[41]	[42]	[15]	Base	Our
Airplane	56.5	76.6	76.1	78.9	**94.1**	80.2	85.2
Bird	66.4	89.5	87.6	**90.9**	69.7	79.5	83.6
Boat	58.0	57.6	62.1	65.9	**88.2**	75.8	79.5
Car	76.8	65.5	80.7	84.8	79.3	86.9	**90.7**
Cat	39.9	43.0	62.4	65.2	76.6	76.5	**78.9**
Cow	69.3	53.4	78.0	81.4	18.6	82.3	**87.4**
Dog	50.4	55.8	58.7	61.9	**89.6**	67.3	71.7
Horse	56.3	37.0	81.8	83.2	**89.0**	85.2	88.1
Moterbike	53.0	24.6	41.5	43.9	**87.3**	58.6	65.8
Train	31.0	62.0	58.2	61.3	75.3	71.7	**77.8**
Mean AP	55.7	56.5	68.7	72.1	76.8	76.4	**80.8**
